# Is bog water chemistry affected by increasing N and S deposition from oil sands development in Northern Alberta, Canada?

**DOI:** 10.1007/s10661-021-09555-4

**Published:** 2021-11-03

**Authors:** R. Kelman Wieder, Melanie A. Vile, Kimberli D. Scott, James C. Quinn, Cara M. Albright, Kelly J. McMillen, Caitlyn Herron, Hope Fillingim

**Affiliations:** 1 Department of Biology, Villanova University, Villanova, PA 19085 USA; 2 Center for Biodiversity and Ecosystem Stewardship, Villanova University, Villanova, PA 19085 USA; 3 Faculty of Science and Technology, Athabasca University, Alberta, T9S 3A3 Canada; 4 Department of Health, West Chester University, West Chester, PA 19383 USA; 5 Department of Geography and the Environment, Villanova University, Villanova, PA 19085 USA; 6 Climate Science Center, Texas Tech University, Lubbock, TX 79409 USA

**Keywords:** Bogs, Deposition, Nitrogen, Sulfur, Oil sands, Porewater

## Abstract

Nitrogen and sulfur emissions from oil sands operations in northern Alberta, Canada have resulted in increasing deposition of N and S to the region’s ecosystems. To assess whether a changing N and S deposition regime affects bog porewater chemistry, we sampled bog porewater at sites at different distances from the oil sands industrial center from 2009 to 2012 (10-cm intervals to a depth of 1 m) and from 2009 to 2019 (top of the bog water table only). We hypothesized that: (1) as atmospheric N and S deposition increases with increasing proximity to the oil sands industrial center, surface porewater concentrations of NH_4_^+^, NO_3_^−^, DON, and SO_4_^2−^ would increase and (2) with increasing N and S deposition, elevated porewater concentrations of NH_4_^+^, NO_3_^−^, DON, and SO_4_^2−^ would be manifested increasingly deeper into the peat profile. We found weak evidence that oil sands N and S emissions affect bog porewater NH_4_^+^-N, NO_3_^−^-N, or DON concentrations. We found mixed evidence that increasing SO_4_^2−^ deposition results in increasing porewater SO_4_^2−^ concentrations. Current SO_4_^2−^ deposition, especially at bogs closest to the oil sands industrial center, likely exceeds the ability of the *Sphagnum* moss layer to retain S through net primary production, such that atmospherically deposited SO_4_^2−^ infiltrates downward into the peat column. Increasing porewater SO_4_^2−^ availability may stimulate dissimilatory sulfate reduction and/or inhibit CH_4_ production, potentially affecting carbon cycling and gaseous fluxes in these bogs.

**Supplementary information:**

The online version contains supplementary material available at 10.1007/s10661-021-09555-4.

## Introduction

Peatlands, ombrotrophic bogs and minerotrophic fens, cover 365,157 km^2^ across northern Alberta, Saskatchewan, and Manitoba, Canada, with non-permafrost bogs covering 27,397 km^2^ of the high boreal and mid-boreal regions (Vitt et al., 2000). Most of these peatlands have persisted over 6000–7000 years (Halsey et al., 2000) in areas of minimal human disturbance in a boreal continental climate with low atmospheric deposition of N and S (< 2 kg N or S ha^−1^ year^−1^). As is typical of bogs worldwide, Alberta bogs have acidic surface porewaters, with low concentrations of dissolved inorganic N, P, S, and base cations (Li & Vitt, 1997; Malmer et al., 1992; Vitt & Wieder, 2007; Vitt et al., 1995; Zoltai & Vitt, 1995).

Oil sands development in northern Alberta has grown markedly since 1967 when the first oil sands mine began operations. Annual emissions of gaseous nitrogen oxides (expressed as NO_2_) from upgrader stacks and diesel-fueled mine fleets have increased over time, reaching about 98,000 metric tonnes in 2019; gaseous SO_2_ emissions peaked in 2009 at about 120,000 metric tonnes and decreased markedly to about 49,000 metric tonnes in 2014, flattening out at about 45,000 metric tonnes through 2019 (updated from Davidson & Spink, 2018; Wieder et al., 2021; Tables S1, S2). These emissions have led to wet and dry N and S deposition that is highest nearest to the oil sands industrial center and decreases with distance (Edgerton et al., 2020; Fenn et al., 2015; Hsu et al., 2016; Wieder et al., 2016a, b).

Increasing N, and to a lesser extent S, deposition has the potential to affect bog ecosystem structure and function (Bobbink & Hettelingh, 2011; Wieder et al., 2016a, b, 2019, 2021; Vitt et al., 2020). Ultimately, when N or S inputs in atmospheric deposition exceed the retention capacity of *Sphagnum* mosses at the bog surface, N and S can move downward into the peat profile (Lamers et al., 2000). One manifestation of depositional inputs exceeding the N, or S, retention capacity is an increase in dissolved inorganic and/or organic N (Yesmin et al., 1995; Williams et al., 1999; Bragazza et al., 2003; Limpens et al., 2003; Tomassen et al., 2003; Bragazza & Limpens, 2004) or sulfate (Gorham et al., 1985; Proctor, 1992; Vile et al., 2003a; Wieder et al., 2016a) concentrations in bog porewaters.

In 2009, we began monitoring bogs in northern Alberta for potential responses to changing atmospheric N and S deposition regimes, with sites situated at different distances from the oil sands industrial center. Bog porewater chemistry was one of the response variables included in the monitoring program. We hypothesized that: (1) as atmospheric N and S deposition increases with increasing proximity to the oil sands industrial center, surface porewater (at the top of the bog water table) concentrations of NH_4_^+^, NO_3_^−^, DON, and SO_4_^2−^ would increase following a threshold response (cf. Lamers et al., 2000) and (2) with increasing N and S deposition, elevated porewater concentrations of NH_4_^+^, NO_3_, DON, and SO_4_^2−^ would be manifested increasingly deeper in the peat profile. To test these hypotheses, we report within-year, between-year, between-site, and depth (10-cm intervals to a depth of 1 m below the peat surface) patterns in porewater concentrations of NH_4_^+^, NO_3_^−^, DON, and SO_4_^2−^ to a depth of 1 m at 5 peatlands from 2009 to 2012, and within-year, between-year, and between-site patterns in surface porewater chemistry at the top of the bog water table at 7 peatlands from 2009 to 2019.

## Methods

### Study sites

**Fig. 1:**
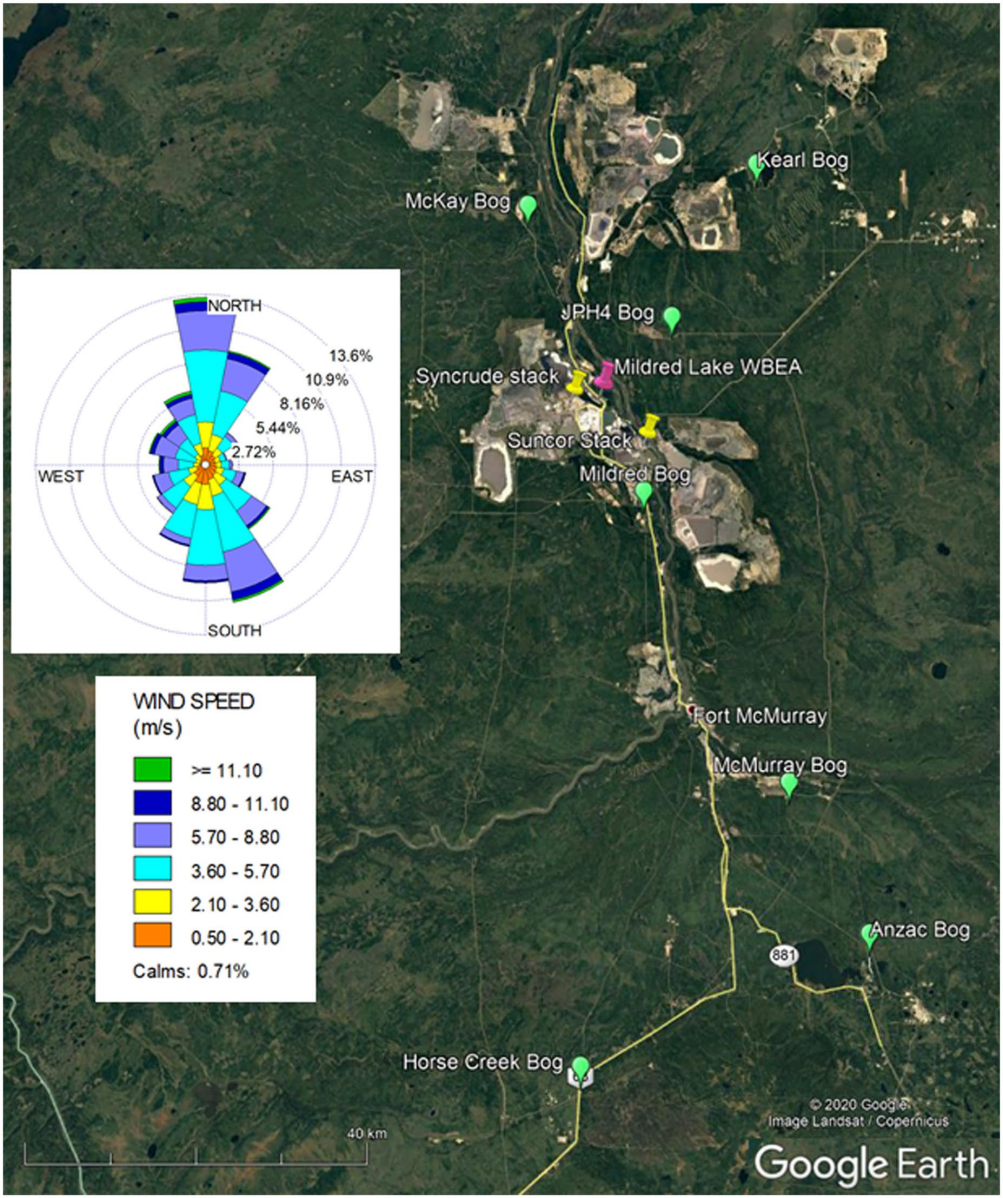
Map of the study region. Bog sites marked with green pins; Syncrude and Suncor upgrader stacks marked with yellow pins; Mildred Lake weather station maintained by the Wood Buffalo Environmental Association marked with a magenta pin. Wind rose based on wind speed and direction at the Mildred Lake weather station, 2009–2019

The peatlands selected for monitoring ranged from 11 to 77 km from the heart of the oil sands mining activity, designated as the midpoint between the Syncrude and Suncor upgrader stacks (Fig. 1). Six of the sites are ombrotrophic bogs, with acidic porewaters (Table 1), a nearly continuous cover of *Sphagnum* mosses (*S. fuscum* and *S. capillifolium* on hummocks, *S. angustifolium* in hollows), and an abundance of *Rhododendron groenlandicum*, *Vaccinium oxycoccos*, and *Vaccinium vitis-idaea*. Porewater at the top of the water table at the Mildred site has a circumneutral pH, although the site has ombrogenous hummocks dominated by *S. fuscum* and/or *S. capillifolium*, and a species composition similar to bogs (Vitt et al., 2020; Wieder et al., 2021). Recognizing the unique nature of the Mildred site, we will refer to all sites as bogs. Four sites were established for the original project in 2009 (Mildred, McMurray, McKay, and Anzac) with a fifth added in 2010 (JPH4). The Mildred site was slated for demolition and was decommissioned in 2013. The Horse Creek and Kearl Lake sites were added for sampling in the 2018 and 2019 seasons.

**Table 1:** Characteristics of the study sites. Weather data from the Interpolated Weather Data Since 1961 for Alberta Townships (https://acis.alberta.ca/township-data-viewer.jsp). Temperature and precipitation values are 30-year means, calculated for the 12-month period of November through October, 1989–2019. NH_4_^+^-N, NO_3_^−^-N, and SO_4_^2−^-S bulk deposition are for 10 years (May 2009–May 2019) from ion exchange resin (IER) collectors replaced in May and October of each year (Vitt et al., 2020; Wieder et al., 2016b). NH_4_^+^-N, NO_3_^−^-N, and SO_4_^2−^-S concentrations in bulk deposition were calculated using total precipitation at each site over the dates when IER collectors were deployed, obtained from Interpolated Weather Data Since 1961 for Alberta Townships (https://acis.alberta.ca/township-data-viewer.jsp)

Parameter	Site						
	Mildred	JPH4	McKay	Kearl	McMurray	Anzac	Horse Creek
Latitude	56°55′49″N	57°6′45″N	57°13′41″N	57°16′21″N	56°37′37″N	56°28′8″N	56°19′45″N
Longitude	111°28′31″W	111°25′23″W	111°42′11″W	111°15′38″W	111°11′44″W	111°2′34″W	111°35′17″W
Years of monitoring	2009–2012	2010–2016 2018–2019	2009–2016 2018–2019	2018–2019	2009–2016 2018–2019	2009–2016 2018–2019	2018 − 2019
Township/range	T092R10W4	T094R09W4	T095R11W4	T95R08W4	T088R08W4	T086R07W4	T084R11W4
Distance from midpoint between Syncrude and Suncor stacks (km)	11	12	24	32	49	69	77
Porewater pH at top of water table	7.04 ± 0.08	3.93 ± 0.03	3.76 ± 0.02	4.69 ± 0.14	3.86 ± 0.01	3.90 ± 0.02	3.86 ± 0.04
Mean annual T (°C)	1.4	1.0	0.9	0.8	1.2	1.1	1.0
Total precipitation (mm/year)	390	412	426	440	417	523	500
NH_4_^+^-N deposition (kg ha^−1^ year^−1^)	0.64 ± 0.04	0.57 ± 0.04	0.61 ± 0.04	0.66 ± 0.11	0.69 ± 0.05	0.76 ± 0.05	0.48 ± 0.08
NO_3_^−^-N deposition (kg ha^−1^ year^−1^)	1.05 ± 0.09	0.75 ± 0.04	0.62 ± 0.03	0.26 ± 0.02	0.60 ± 0.03	0.53 ± 0.02	0.27 ± 0.02
SO_4_^2−^-S deposition (kg ha^−1^ year^−1^)	9.02 ± 0.78	9.88 ± 0.93	7.69 ± 0.74	3.11 ± 0.85	4.26 ± 0.31	2.88 ± 0.20	1.66 ± 0.42
NH_4_^+^-N in precipitation (mg/L)	0.22 ± 0.02	0.17 ± 0.01	0.18 ± 0.01	0.23 ± 0.04	0.25 ± 0.03	0.22 ± 0.02	0.26 ± 0.05
NO_3_^−^-N in precipitation (mg/L)	0.43 ± 0.03	0.29 ± 0.02	0.25 ± 0.01	0.08 ± 0.01	0.25 ± 0.02	0.23 ± 0.02	0.12 ± 0.02
SO_4_^2−^-S in precipitation (mg/L)	4.12 ± 0.40	4.20 ± 0.43	2.95 ± 0.25	1.20 ± 0.40	1.72 ± 0.12	1.16 ± 0.09	0.80 ± 0.27

### Weather

Mean daily temperature and daily precipitation were obtained for each site from 1989 to 2019 from Interpolated Weather Data Since 1961 for Alberta Townships (https://acis.alberta.ca/township-data-viewer.jsp). To assess meteorological drought conditions over the 30-year study period, we used the standardized precipitation index, using data from the Alberta government Mildred Lake Station (57.03°N, 111.45°W; https://acis.alberta.ca/weather-data-viewer.jsp) and the DrinC package (Tigkas et al., 2015, www.drought-software.com). Wind roses were generated using wind speed and wind direction data from the Mildred Lake Station using WRPLOT View, version 8.0.2 (Lakes Environmental Software, Waterloo, Ontario, Canada).

### Sampling and analysis

At each site, we installed three, 1-m long porewater samplers, each consisting of 10 10-cm sections of thinly slotted 2.5 cm-diameter PVC pipe, with sections separated from each other by a polypropylene disk. Color-coded Tygon® tubing extended from each depth section to the peat surface. The samplers allowed us to collect peat porewater from discrete 10-cm depth intervals from the top of the water table to 1 m below the peat surface. Using a 60-mL syringe, we evacuated and discarded porewater from each depth and allowed the porewater sampler segments to passively refill with porewater prior to sample collection. Porewater was collected from each depth segment using a syringe and was field filtered (Whatman 41 filter paper) into acid-washed Nalgene bottles. Sampling at the original set of sites occurred on multiple dates between May and October from 2009 to 2012. Samples from the Mildred site for August through October 2011 were destroyed in the laboratory fire at the Meanook Biological Research Station. Porewater sampling to a depth of 1 m was discontinued after the 2012 field season for all sites; porewater sampling at the top of the peatland water table continued through 2019.

In the laboratory, porewater samples were analyzed for pH, NH_4_^+^-N (phenate method, Seal AA3 AutoAnalyzer), NO_3_^−^-N, and SO_4_^2−^-S (DIONEX ion chromatograph), and DON (dissolved organic nitrogen) (Shimadzu TOC-V_CSH_ analyzer and TNM-1 total nitrogen detector, with prefiltration through 0.45-μm filters).

To simplify visual presentation of the 2009–2012 data, we prepared 2-dimensional plots showing changes in porewater chemical parameters as a function of depth below the water table and time using PROC G3GRID in SAS with spline interpolation, a smoothing factor of 0.05, and gridding at 5-cm depth intervals and 15-day time intervals. Interpolation was temporally bounded by the first and last sampling date of each year. Resulting grid data were used to generate contour plots in SigmaPlot 14.0. The position of the water table was inferred from the topmost segment of the samplers from which water could be collected on a given sampling date.

## Results

### Weather

Over the 30-year period from 1989 to 2019, mean annual temperature averaged 1.1 °C and total annual precipitation averaged 445 mm year^−1^ (Table 1). Over this 30-year period, mean annual temperature increased at a rate of 0.036 °C year^−1^ (*p* = 0.0002) and total annual precipitation decreased at a rate of 1.69 mm year^−1^ (*p* = 0.0199); rates were consistent across all sites (*p* values for year by site interactions were 0.5400 and 0.9406, respectively; analysis of covariance for homogeneity of slopes). Site comparisons indicated that on average, Mildred was the warmest (mean annual temperature 1.4 °C), while Kearl was the coolest (mean annual temperature 0.8 °C) (Table S3). Anzac and Horse Creek had the highest annual precipitation (512 mm year^−1^); Mildred had the lowest (399 mm year^−1^), and Anzac the wettest (Table S3). Weather conditions were quite variable from year to year. Averaged across all sites, the 11-year monitoring period included relatively warm years (2014–2015, 2010–2011, 2015–2016, and 2008–2009 ranked the 3rd, 4th 5th, and 6th warmest) and relatively cool years (2017–2018, 2016–2017, and 2012–2013 ranked the 21st, 22nd, and 29th coolest), as well as relatively wet years (2001–2012, 2015–2016, and 2012–2013 ranked the 2nd, 6th, and 8th wettest years) and relatively dry years (2008–2009, 2009–2010, 2010–2011, 2014–2015, and 2016–2017 ranked the 22nd, 25th, 27th, 28th, and 29th driest years) (Table S3).

**Fig. 2:**
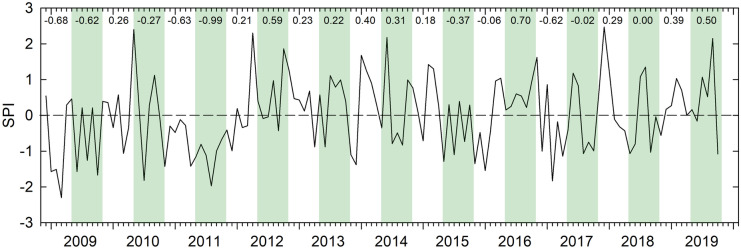
Standardized precipitation index (SPI; Guttman, 1999; McKee et al., 1993), calculated using data from the Alberta government Mildred Lake Station. Green shaded regions indicate growing season months (May–October). Numbers across the top are mean SPI values within a given 6-month period

The standardized precipitation index (SPI; Guttman, 1999; McKee et al., 1993), an algorithm that calculates the number of standard deviations that observed precipitation deviates from the climatological average, also showed considerable variability over the 11-year monitoring period (Fig. 2). Of particular note is that the 2011 growing season had SPI values that were less than − 1 for every month, and this was preceded by a 6-month non-growing season in which monthly SPI values were negative in each month, indicating a year of relatively dry conditions.

### N and S in bulk deposition

Across the seven sites, NO_3_^−^-N and SO_4_^2−^-S, but not NH_4_^+^-N, bulk deposition (kg ha year^-1^) and concentrations (mg L^-1^) in bulk deposition, show a general pattern of decreasing with increasing distance from the oil sands industrial center (Table 1).

### Porewater chemistry throughout the top 1 m of peat, 2009–2012

**Fig. 3:**
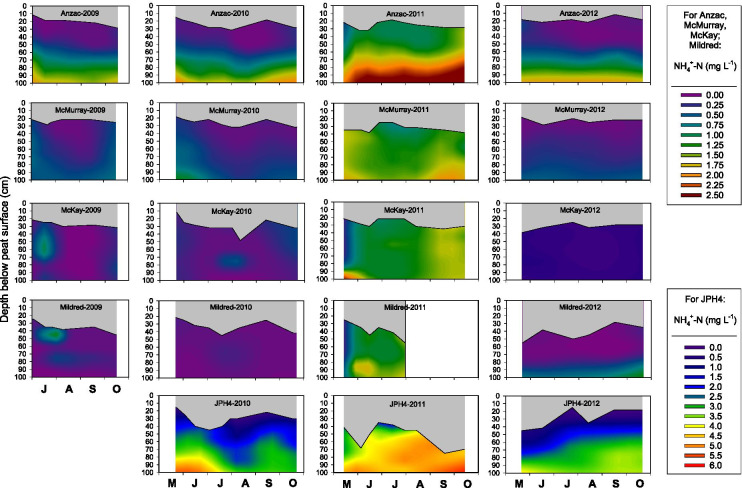
Interpolated porewater NH_4_^+^-N concentrations at 5 peatland sites as a function of time and depth. Gray areas indicate the zone of peat between the peat surface and the water table

Porewater NH_4_^+^-N concentrations varied between sites, years, and depth (Fig. 3), exhibiting significant site by year (*p* = 0.0470) and site by depth (*p* < 0.0001) interactions (Tables S4, S5, S6). Although NH_4_^+^-N concentrations were highest at JPH4 from 2010 to 2012, there was no clear pattern of NH_4_^+^-N concentrations decreasing with distance from the oil sands industrial center (Fig. 3, Tables S5, S6). At all 5 sites, porewater NH_4_^+^-N concentrations were substantially higher in 2011 than in the other 3 years (Table S5). In general, NH_4_^+^-N concentrations increased with depth, although this increase was more pronounced at JPH4, McKay, and Anzac than at Mildred or McMurray (Fig. 3, Table S6). Although porewater NH_4_^+^-N concentrations exhibited some within-year seasonal variation, patterns were not consistent across all sites (Fig. 3).

**Fig. 4:**
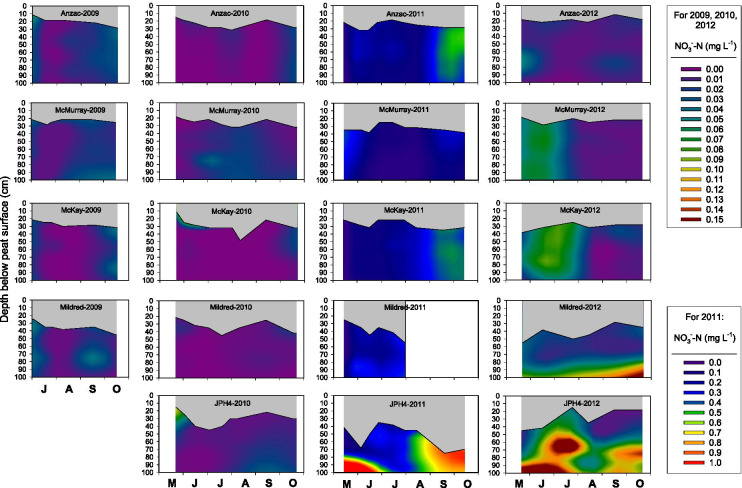
Interpolated porewater NO_3_^−^-N concentrations at 5 peatland sites as a function of time and depth. Gray areas indicate the zone of peat between the peat surface and the water table

Porewater NO_3_^−^-N concentrations varied between sites, years, and depth (Fig. 4), exhibiting significant site by year (*p* < 0.0001) and year by depth (*p* = 0.0111) interactions (Tables S4, S7, S8). There was no clear pattern of NO_3_^−^-N concentrations decreasing with distance from the oil sands industrial center (Table S7). At all sites, porewater NO_3_^−^-N concentrations were substantially higher in 2011 than in the other 3 years (Table S7). No clear pattern of changing porewater NO_3_^−^-N concentrations with depth was observed at any of the sites; porewater NO_3_^−^-N concentrations in the uppermost peat did not decrease with increasing distance from the oil sands industrial center (Fig. 4, Tables S7, S8). Although there was within-year seasonal variation in NO_3_^−^-N concentrations, these patterns were not consistent across all sites (Fig. 4). Averaged across all sites, depths, and sampling dates, porewater NO_3_^−^-N concentrations were equivalent to 14% of porewater NH_4_^+^-N concentrations.

**Fig. 5:**
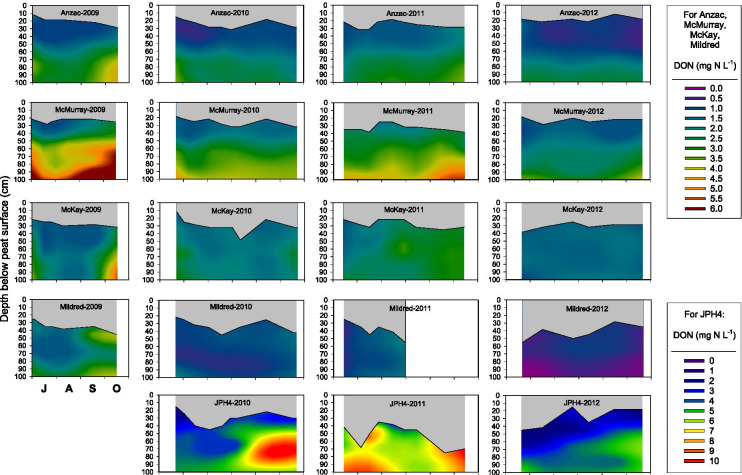
Interpolated porewater DON concentrations at 5 peatland sites as a function of time and depth. Gray areas indicate the zone of peat between the peat surface and the water table

Porewater DON concentrations varied between sites, years, and depth (Fig. 5), exhibiting significant site by year (*p* < 0.0001), site by depth (*p* < 0.0001), and year by depth (*p* = 0.0111) interactions (Tables S4, S9, S10, S11). Although DON concentrations were highest at JPH4 from 2010 to 2012, there was no clear pattern of DON concentrations decreasing with distance from the oil sands industrial center (Fig. 5, Tables S9, S10). For all sites except Mildred, DON concentrations increased with depth, with the increase being most pronounced at JPH4 and least pronounced at McKay and Anzac (Fig. 4, Table S10). Increases in DON concentration with depth were slightly more evident in 2009 and 2011 than in 2010 and 2012 (Fig. 5, Table S11). Within-year seasonal variation in DON concentrations was not consistent across all of the sites (Fig. 5).

Averaged across all sites, depths, and sampling dates, porewater DON concentrations were equivalent to 77% of porewater total dissolved N (TDN) concentrations. The DON:TDN ratio exhibited a significant site by year by depth interaction (Tables S4, S12). Generally, DON/TDN ratios were lower in 2011 (60.6 ± 0.4, *n* = 625) than in the other 3 years (86.0 ± 0.6, *n* = 464; 83.4 ± 0.5, *n* = 655; 79.9 ± 0.7, *n* = 513 in 2009, 2010, 2012, respectively).

**Fig. 6:**
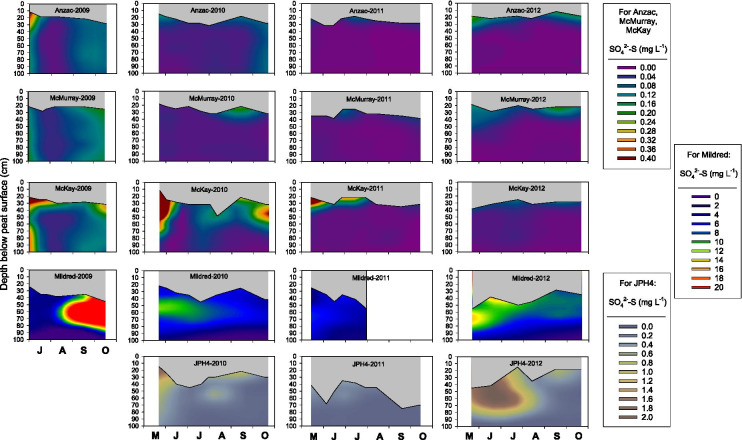
Interpolated porewater SO_4_^2−^-S concentrations at 5 peatland sites as a function of time and depth. Gray areas indicate the zone of peat between the peat surface and the water table

Porewater SO_4_^2−^-S concentrations varied between sites, years, and depth (Fig. 6), exhibiting a significant site by year by depth interaction (Tables S4, S14). The five peatland sites differed considerably with respect to porewater SO_4_^2−^-S concentrations. Averaged across all sampling dates (2009–2012) and depths, mean SO_4_^2−^-S concentrations at Mildred, JPH4, McKay, McMurray, and Anzac were 6667 ± 294, 582 ± 61, 184 ± 18, 42 ± 4, and 39 ± 2 µg L^−1^; thus, SO_4_^2−^-S concentrations decreased with increasing distance from the oil sands industrial center. Variation in porewater SO_4_^2−^-S concentration with depth and within a given year was not consistent between sites (Fig. 6).

### Porewater chemistry, top of the water table, 2009–2019

**Fig. 7:**
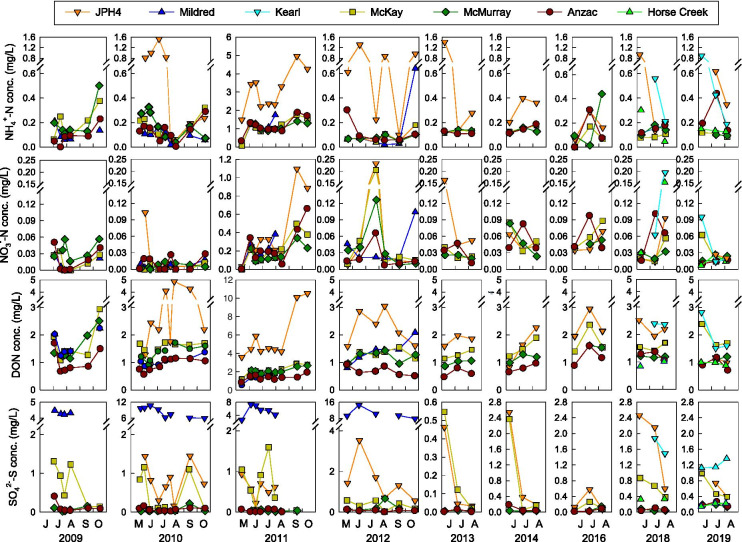
Porewater NH_4_^+^-N, NO_3_^−^-N, DON, and SO_4_^2−^-S concentrations at the top of the peatland water table at 7 peatland sites, 2009–2019. Values are means (*n* = 3)

**Fig. 8:**
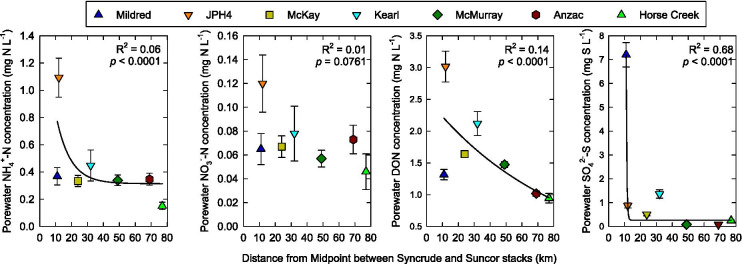
Porewater NH_4_^+^-N, NO_3_^−^-N, DON, and SO_4_^2−^-S concentrations, averaged across all sampling dates in all years

Porewater concentrations of NH_4_^+^-N, NO_3_^−^-N, DON, and SO_4_^2−^-S at the top of the peatland water table were variable within years, between years (2009–2019), and between sites (Fig. 7). Over the 11-year sampling period, mean NH_4_^+^-N, NO_3_^−^-N, DON, and SO_4_^2−^-S concentrations, averaged across all sampling dates in all years, decreased exponentially with distance from the midpoint between the Syncrude and Suncor stacks (*p* ≤ 0.0761), although *R*^2^ values were low; ≤ 0.14 for NH_4_^+^-N, NO_3_^−^-N, and DON concentrations (Fig. 8). At three of the sites, porewater NO_3_^−^-N concentrations increased with time since the initial sampling in 2009, porewater NH_4_^+^-N, DON, and SO_4_^2−^-S concentrations were not correlated with time (Table 2). Across all sites and years, for the most part, porewater NH_4_^+^-N, NO_3_^−^-N, and DON concentrations were positively correlated with both growing season NH_4_^+^-N and NO_3_^−^-N concentrations in precipitation and growing season NH_4_^+^-N and NO_3_^−^-N deposition (Table 3). Porewater SO_4_^2−^-S concentrations were positively correlated with both growing season SO_4_^2−^-S concentration in precipitation and growing season SO_4_^2−^-S deposition (Table 3).

**Table 2:** Kendall’s correlations (τ correlation coefficient and associated *p* values) between porewater concentrations of NH_4_^+^-N, NO_3_^−^-N, DON, and SO_4_^2−^-S at the top of the peatland water table and time (days since the first sampling date in 2009). Significant positive correlations (one-sided test) of dissolved N components and negative correlations (one-sided test) with SO_4_^2−^-S are highlighted in bold, italic font. The Kearl and Horse Creek sites are not included as sampling began in 2018, so data are temporally restricted

Porewater constituent	Kendall’s τ parameters	Site				
		Mildred	JPH4	McKay	McMurray	Anzac
NH_4_^+^-N	τ	0.0273	− 0.1857	− 0.1035	− *0.1648*	0.0881
	*p*	0.7464	0.0102	0.0861	*0.0082*	0.1392
NO_3_^−^-N	τ	***0.3369***	− 0.0002	***0.2017***	0.0996	***0.1886***
	*p*	***0.0001***	0.9972	***0.0010***	0.1120	***0.0019***
DON	τ	0.0030	− 0.3134	0.0488	− 0.1697	0.0036
	*p*	0.9722	< 0.0001	0.4276	0.0076	0.9518
SO_4_^2−^-S	τ	0.2098	− 0.0329	− 0.0942	0.0819	− 0.0425
	*p*	0.0148	0.6545	0.1272	0.2017	0.4684

**Table 3:** Kendall’s τ correlation coefficients and associated *p* values between inorganic N and S in peatland porewater at the top of the water table and growing season inorganic N and S concentration in precipitation and deposition, 2009–2019. Significant correlations are shown in bold, italic font

Concentration in porewater	Concentration in precipitation (mg L^−1^)			Growing season deposition (kg ha^−1^)		
	NH_4_^+^-N	NO_3_^−^-N	SO_4_^2−^-S	NH_4_^+^-N	NO_3_^−^-N	SO_4_^2−^-S
NH_4_^+^-N	***0.0852*** ***0.0028***	***0.2015*** ** < *0.0001***		− 0.0016 0.9549	***0.1763*** ** < *0.0001***	
NO_3_^−^-N	***0.1530*** ** < *0.0001***	***0.2728*** ** < *0.0001***		***0.2144*** ** < *0.0001***	***0.3384*** ** < *0.0001***	
DON	***0.1158*** ** < *0.0001***	***0.2391*** ** < *0.0001***		***0.0085*** ***0.7705***	***0.2279*** ** < *0.0001***	
SO_4_^2−^-S			***0.2556*** ** < *0.0001***			***0.1211*** ** < *0.0001***

## Discussion

### Porewater chemistry with depth over time, 2009–2012

Over the first 4 years of sampling, we found little evidence to support either of our hypotheses especially for N. Porewater concentrations of NH_4_^+^, NO_3_^−^, and DON at the top of the peatland water table did not clearly decrease with increasing distance from the oil sands industrial center. We also found no evidence to support that with increasing deposition, elevated concentrations of NH_4_^+^, NO_3_^−^, DON, and SO_4_^2−^ would be manifested deeper in the peat profile. Porewater NH_4_^+^, DON, and SO_4_^2−^ concentrations, however, did decrease with increasing distance from the oil sands industrial center.

The pattern of increasing NH_4_^+^-N and DON, but not NO_3_^−^-N, concentrations with depth, observed across all sites, is consistent with findings from other boreal or north temperate bogs (e.g., Bleak Lake Bog, Alberta, Vitt et al., 1995; S1 bog in the Marcell Experimental Forest (MEF), Minnesota, Griffiths & Sebestyen, 2016), but this pattern has not been evident at all bogs (e.g., Mer Bleue, Ontario, Basiliko et al., 2005). Total soluble N concentrations increased with depth (up to 3 m) at the S1 bog in the MEF consistently over 3 years of measurement (Griffiths & Sebestyen, 2016).

Few studies have quantified porewater SO_4_^2−^-S concentrations as a function of depth in bogs. At Bleak Lake Bog, Alberta, porewater SO_4_^2−^-S concentrations were either uniform of decreased over the top 20 cm of peat; S-amended plots (25.6 kg S ha^−1^ year^−1^) had higher porewater SO_4_^2−^-S concentrations than control plots (Vile et al., 2003a). At the S2 bog in the MEF, porewater SO_4_^2−^-S concentrations were either uniform, or slightly higher near the top of the water table than deeper in 35–50 cm peat profiles (Urban et al., 1989). In 9 temperate peatlands in eastern North America and the Czech Republic, porewater SO_4_^2−^-S concentrations were either uniform across the top 45 cm of peat or higher in the top 5 cm of peat and uniform from 5 to 45 cm (Novák & Wieder, 1992). While the biogeochemical processes that affect N and S cycling in bogs are generally known (cf. Blodau et al., 2007; Limpens et al., 2006; Vile & Novák, 2006), how these processes generate the considerable variability in depth profiles of soluble inorganic N and S within sites, between sites, and between years is not yet fully understood.

One of the most striking findings from our study is the considerably higher concentration of porewater NH_4_^+^ and NO_3_^−^, but not of DON or SO_4_^2−^, throughout the top 1 m of peat in 2011 relative to the other 3 years (Figs. 3–7), a pattern that we also observed in porewater at the top of the water table in control plots (receiving no added N) in a bog and poor fen near Mariana Lake, Alberta, 115 km south of oil sands industrial center (Wieder et al., 2019, 2020). The 2010–2011 water year was unusually warm and dry (Table S3). Moreover, the 2011 growing season was unusually dry and was preceded by an unusually dry winter (Fig. 2). It is possible that under these conditions, N mineralization in near-surface peat could have been stimulated by warmer than normal surface peat, leading to the anomalously high porewater NH_4_^+^-N and NO_3_^−^-N concentrations. However, it seems unlikely that N mineralization would have been stimulated in deeper peat as temperature variation is dampened with depth in peat, and where water-saturated conditions prevail below the bog water table. Yet in 2011, we observed elevated NH_4_^+^-N and NO_3_^−^-N concentrations throughout the water-saturated peat to a depth of 1 m (Fig. 3). Even if the unusually warm and dry conditions at our monitoring sites had stimulated N mineralization in unsaturated near-surface peat, the NH_4_^+^-N and NO_3_^−^-N produced by mineralization is likely to have been immobilized by cation exchange (for NH_4_^+^-N) and plant/microbial uptake rather than moving downward into the saturated peat (cf. Wieder et al., 2019, 2020). Indeed, in the control plots at both the Mariana Lake bog and poor fen, leaf N concentrations in *Andromeda polifolia* and *Chamaedaphne calyculata* (but not in leaves of other major vascular plant species) and N concentrations in *Sphagnum fuscum* stems were higher in 2011 than in the four subsequent years (Wieder et al., 2019, 2020). However, we note that in the control plots of the Mariana Lake bog and poor fen, neither net N mineralization (net ammonification, net nitrification, and net DON production) nor KCl-extractable NH_4_^+^-N and NO_3_^−^-N concentrations in the top 10 cm of peat below the living *Sphagnum* layer were elevated in 2011 as compared to 2012–2015 (Wieder et al., 2019, 2020). Thus, it appears that stimulation of N mineralization by the relatively warm, dry conditions of the 2011 growing season is not sufficient to explain the anomalously high elevated NH_4_^+^-N and NO_3_^−^-N concentrations that we observed at all monitoring sites in bog porewater below the water table.

At the bog monitoring sites, mean water table position, averaged across all sites and measurement dates, was 6 cm lower in 2011 than in 2012–2015 (Figs. 3–6). During drought conditions, a decreased hydraulic head in bogs can lead to hydraulic reversals such that peatland porewater may not be completely isolated from deeper groundwater (DeVito et al., 1997; Romanowicz et al., 1993; Ulanowski & Branfireun, 2013). Thus, another possible explanation of the anomalously high porewater NH_4_^+^ and NO_3_^−^ concentrations in 2011 could be a hydraulic reversal such that bog porewater chemistry was influenced by deep bog porewater and/or groundwater with higher NH_4_^+^-N and NO_3_^−^-N concentrations than bog porewater throughout the upper 1 m of peat in normal years. We acknowledge, however, that at this point, we cannot offer a definitive mechanistic explanation for the high NH_4_^+^-N and NO_3_^−^-N concentrations in 2011 observed at all bog monitoring sites, throughout the top 1 m of peat, over the entire growing season.

### Temporal changes in porewater chemistry at the top of the bog water table, 2009–2019

Over the 11-year period, concentrations of NH_4_^+^-N, NO_3_^−^-N, DON, and SO_4_^2−^-S at the top of the peatland water table averaged 460 ± 31, 74 ± 6, 1629 ± 51, and 1197 ± 116 µg L^−1^, respectively. Although there was within-site variability for each of these porewater constituents, this variability was not consistent across sites and/or years. Our measured values for NH_4_^+^-N, NO_3_^−^-N, DON, and SO_4_^2−^-S are within the ranges reported for porewater in surface pools or at the top of the peatland water table for other boreal bogs across Canada (Table 4).

**Table 4:** Surface (top of the bog water table or surface pools) porewater concentrations of NH_4_^+^-N, NO_3_^−^-N, DON, and SO_4_^2−^-S in boreal Canadian bogs. Values are means ± either standard deviations or standard errors, ranges; or a single mean value when neither uncertainties nor ranges were reported. Sites are arranged from west to east across Canada

Bog site	Latitude, longitude	NH_4_^+^-N (µg L^−1^)	NO_3_^−^-N (µg L^−1^)	DON (µg L^−1^)	SO_4_^2−^-S (µg L^−1^)	Ref
Graham Island, BC Banks Island, BC Baron Island, BC	53°58′N, 131°45′W 53°25′N, 130°17′W 54°28′N, 130°49′W				900–2500	1
Big Bay, BC McCauley Island, BC Farrant Island, BC	54°27′N, 130°26′W 53°41′N, 130°15′W 53°22′N, 129°25′W				600–1800	1
Seba Beach, AB	53°33′N, 114°44′W	100	0		900	2
Bleak Lake Bog, AB, 1989 Bleak Lake Bog, AB, 1980	54°41′N, 113°28′W	18 ± 8 17 ± 19	6 ± 2 11 ± 7			3
Bleak Lake Bog, AB	54°41′N, 113°28′W	9–84	1–8			4
Bleak Lake Bog, AB	54°41′N, 113°28′W				2240	5
21 bog sites, AB	Oil sands region	1220–1740	90–200	1910–3350	10–2690	6
Mariana Lake Bog, AB, 2011 Mariana Lake Bog, AB, 2012 Mariana Lake Bog, AB, 2013 Mariana Lake Bog, AB, 2014 Mariana Lake Bog, AB, 2015	55°53′N, 112°16′W	1370 ± 100 100 ± 10 120 ± 10 130 ± 30 220 ± 20	180 ± 30 3 ± 1 10 ± 4 20 ± 8 11 ± 3	1603 ± 165 1029 ± 35 1016 ± 75 1281 ± 60 750 ± 169		7
Mire 239, Experimental Lakes Area, ON	49°40′N, 93°43′W	16 ± 15			247 ± 213	8
Hudson Bay Lowlands, July Hudson Bay Lowlands, August	52.821 N, 83.884 W				97 ± 77 23 ± 17	9
Hudson Bay Lowlands, 12 bogs	51°N, 82–84°W		0		6–567	10
Mer Bleue, ON (Control plots)	45.40°N, 75.50°W	43 ± 21	84 ± 35	3731 ± 854		11
Saint-Marguerite-Marie, QC	48°47′N, 72°10′W	200	< 100		867	2
Maisonette, NB	47°49′N, 65°02′W	1600	< 100		633	2
Rivière-Ouelle, QC	47°27′N, 69°56′W	< 100	60		3200	2

^1^Malmer et al. (1992), ^2^Wind-Mulder et al. (1996), ^3^Vitt et al. (1995), ^4^Szumigalski and Bayley (1997), ^5^Vile et al. (2003a), ^6^Wieder et al. (2016a), ^7^Wieder et al. (2019), ^8^Vitt and Bayley (1984), ^9^Ulanowski and Branfireun (2013), ^10^Glaser et al. (2004), ^11^Xing et al. (2011)

Three lines of evidence would support the hypothesis that porewater NH_4_^+^-N, NO_3_^−^-N, DON, and SO_4_^2−^-S concentrations at the top of the peatland water are influenced by N and S emissions from oil sands operations. First, porewater concentrations should decrease exponentially with distance from the oil sands industrial center. Second, over the 11-year period, porewater NH_4_^+^-N, NO_3_^−^-N, and DON concentrations should increase and porewater SO_4_^2−^-S concentrations should decrease over time, corresponding to increasing N and decreasing S oil sands emissions (Davidson & Spink, 2018; Wieder et al., 2021, Tables S1, S2). Third, porewater NH_4_^+^-N, NO_3_^−^-N, and DON concentrations should be correlated with growing season NH_4_^+^-N and/or NO_3_^−^-N concentrations in precipitation or deposition, and porewater SO_4_^2−^-S concentrations should be correlated with growing season SO_4_^2−^-S deposition.

Concentrations of NH_4_^+^-N, DON, and SO_4_^2−^-S, but not NO_3_^−^-N decreased exponentially with distance from the oil sands industrial center (midpoint between the Syncrude and Suncor stacks), albeit with quite low *R*^2^ values for NH_4_^+^-N and DON (Fig. 8). Exponential decreases in concentrations with distance would be consistent with oil sands N and S emissions influencing porewater chemistry. For porewater NH_4_^+^-N, NO_3_^−^-N, and DON concentrations, these spatial patterns are strongly driven by high concentrations in porewater at the JPH4 site. For porewater SO_4_^2−^-S concentration, the decrease with distance was strongly driven by high concentrations at the Mildred site. Prevailing winds were from the south or south southeast 20% of the time over the 11-year period, carrying N and S emissions toward JPH4; prevailing winds were from the north or north northeast 22% of the time, toward the Mildred site (Fig. 1). These patterns were consistent across all years (Fig. S1). If wind-dispersion of N and S emissions was the primary cause of decreasing porewater concentrations with distance, we would expect high porewater concentrations of NH_4_^+^-N, NO_3_^−^-N, DON, and SO_4_^2−^-S at both JPH4 and Mildred, which was not the case. It is possible that the unusually high SO_4_^2−^-S concentrations at the Mildred site are, at least in part, the result of upward diffusion or advection of shallow groundwater from weathered tills with high SO_4_^2−^ concentrations, known to occur in the oil sands region (Birks et al., 2019; Cowie et al., 2015). At the Mildred site, porewater reduced electrical conductivity (Sjörs, 1952) increased considerably with depth throughout the upper 1 m of peat, reaching values as high as 1000 µS cm^−1^ (Fig. S2), a value higher than found in extreme rich fens in Canada (cf. Gignac et al., 2004; Hartsock et al., 2019a; Slack et al., 1980; Vitt & Chee, 1990; Vitt et al., 1995), but lower than in saline fens in northern Alberta (cf. Hartsock et al., 2019b; Volik et al., 2017).

From 2009 through 2019, oil sands stack and fleet N emissions have steadily increased (from 33,437 to 97,873 metric tonnes year^−1^), while S emissions have dramatically decreased from 2009 through 2014 (from 120,052 to 48,923 metric tonnes year^−1^), and remaining relatively constant at about 45,000 metric tonnes year^−1^ through 2019. If oil sands emissions are influencing bog porewater chemistry, we would expect to see corresponding increases in porewater NH_4_^+^-N, NO_3_^−^-N, and/or DON concentrations, and decreases in porewater SO_4_^2−^-S concentrations over the 11-year sampling period. While porewater NO_3_^−^-N concentrations did increase over time at three of the sites (Mildred, McKay, and Anzac, Table 2), concentrations of NH_4_^+^-N, DON, or SO_4_^2−^-S showed no significant directional change over the 11-year period. This metric does not provide strong support for N and S emissions affecting peatland porewater chemistry.

We did find, however, that at the top of the bog water table, porewater NH_4_^+^-N, NO_3_^−^-N, DON concentrations and SO_4_^2−^-S concentrations were significantly correlated with growing season N and S deposition (quantified using ion exchange resin collectors), respectively, at each site and with calculated N and S concentrations in precipitation (Table 3). It is well established that wet and dry N and S deposition decrease with increasing distance from the oil sands industrial center (Edgerton et al., 2020; Fenn et al., 2015; Hsu et al., 2016; Wieder et al., 2016a, b). A one-time synoptic sampling of 19 bogs distributed across a 3,255 km^2^ area centered on the midpoint between the Syncrude and Suncor stacks revealed significant correlations of bog porewater SO_4_^2−^-S with interpolated SO_4_^2−^-S deposition, but no significant correlations of porewater NH_4_^+^-N, NO_3_^−^-N, or DON with NH_4_^+^-N or NO_3_^−^-N deposition (Wieder et al., 2016a). All of these factors taken together suggest that bog porewater SO_4_^2−^-S concentrations may be more strongly affected by oil sands S emissions than porewater NH_4_^+^-N, NO_3_^−^-N, or DON concentrations to N emissions.

Annual net primary production (NPP) of *Sphagnum fuscum* across 6 bogs, 5 of which are the same as bogs used in the present study, was 259 ± 9 g dry mass m^−2^ year^−1^ (Wieder et al., 2016b). At these bogs, S concentrations in *S. fuscum* capitula ranged from 1.13 to 1.83 mg g^−1^ and decreased with increasing distance from the oil sands industrial center (Wieder et al., 2021). Therefore, the annual quantity of S taken up through NPP of *S. fuscum* would be 2.9–4.7 kg ha^−1^ year^−1^. Especially at the bog sites closest to the oil sands industrial center, atmospheric SO_4_^2−^-S deposition (Table 1) is at least sufficient to support uptake by growing *S. fuscum*, and dry deposition of S would supply even more S than measured using ion exchange resin collectors (cf. Edgerton et al., 2020). Thus, the *Sphagnum* “filter” for S may have failed, leading to downward movement of atmospherically deposited SO_4_^2−^-S into the peat profile, affecting porewater SO_4_^2−^-S concentrations. Some of the SO_4_^2−^ not used for *Sphagnum* growth is taken up by vascular plants. However, if S deposition is sufficient to cause the downward infiltration of sulfate-enriched into the peat profile, dissimilatory sulfate reduction could be stimulated, changing the pathways of peat decomposition that ultimately produce and release CO_2_ to the atmosphere (Vile et al., 2003b). At the same time, in bogs generally, increasing SO_4_^2−^-S deposition leads to a strong suppression of CH_4_ fluxes to the atmosphere (Gauci et al., 2004). Therefore, increasing SO_4_^2−^-S deposition above background levels in the oil sands region may have broader consequences with respect to bog CO_2_ and CH_4_ emissions.

### Comparisons with European studies

Several field or greenhouse studies in European bogs have shown strong evidence that NH_4_^+^-N, NO_3_^−^-N, and/or DON concentrations in surface pools or in porewater at the top of the bog water table increase with increasing N deposition, either along prevailing N and S deposition gradients or with experimental N addition (Bragazza & Limpens, 2004; Bragazza et al., 2003; Limpens et al., 2003; Tomassen et al., 2003; Williams et al., 1999; Yesmin et al., 1995). However, background N deposition at these bog sites in general was considerably higher (up to 35 kg N ha^−1^ year^−1^) than deposition in the oil sands region, and these high deposition levels may have persisted for decades. Further, for the European studies that included experimental N addition in the greenhouse or in the field, N treatments have often been at exceptionally high levels (up to 100 kg N ha^−1^ year^−1^). Thus, given the background and experimental N addition conditions in these European studies, their results may not have clear relevance to conditions in the oil sands region.

When NH_4_NO_3_ was added to bog peat cores collected from a low N deposition site (Mariana Lake Bog, Alberta; 1.7 kg N ha^−1^ year^−1^) at rates up to 45 kg N ha^−1^ year^−1^, consistently across all N treatment levels, 89% of the added NH_4_^+^-N and 50% of the added NO_3_^−^-N were retained in the peat, with no effect on DON leaching (Hartsock et al., 2018). When NH_4_NO_3_ was added in the field to Mariana Lake Bog, 115 km south of the oil sands, region over a 4-year period at levels up to 25 kg N ha^−1^ year^−1^, no increase in porewater NH_4_^+^-N, NO_3_^−^-N, or DON was observed (Wieder et al., 2019). The absence of a porewater chemistry response in these instances may be related to the important role of biological N_2_-fixation in Alberta bogs (Vile et al., 2014). At Mariana Lake Bog, field NH_4_NO_3_ addition caused a downregulation of N_2_-fixation that was similar in magnitude to the quantity of N added, such that the result was no change in the total amount of N added to the bog through both deposition and fixation (Wieder et al., 2019). None of the European studies mentioned above considered N_2_-fixation, but it is likely the case that with chronic high background N deposition, N_2_-fixation is of minimal importance at many of these bogs. Based on results from Mariana Lake Bog, and the bog monitoring sites in this study, N deposition rates today (Table 1) are not high enough to completely inhibit N_2_-fixation. However, if N emissions from oil sands operations continue to increase, N_2_-fixation should progressively decrease, which could eventually lead to increasing NH_4_^+^-N, NO_3_^−^-N, and/or DON concentrations in bog porewaters.

Of the European studies, two showed increasing porewater DON concentrations with increasing N deposition (Bragazza & Limpens, 2004; Williams et al., 1999). Bragazza and Limpens (2004) showed clear, linear, increases in both DIN and DON in bogs along a N deposition gradient of 2–20 kg N ha^−1^ year^−1^. Along the gradient, DON concentrations were approximately 3 times higher than DIN concentrations at each site, with DON concentrations increasing to 65 µg L^−1^ at the highest N deposition site. The DON response was interpreted as resulting from the uptake of atmospherically deposited N by *Sphagnum*, its incorporation into amino acids, and the release of these organic N molecules into bog porewater (Bragazza & Limpens, 2004). It is unclear whether this sequence of events occurs in Alberta bogs, where *Sphagnum* capitulum N concentrations show only small increases in response to N deposition either at the bog monitoring sites (Table 1) (Wieder et al., 2021) or with experimental field N addition (Wieder et al., 2019), where porewater DON concentrations in general are substantially higher (Fig. 5) than those reported by Bragazza and Limpens (2004).

## Conclusions

We found only weak evidence that bog porewater NH_4_^+^-N, NO_3_^−^-N, or DON concentrations at the top of the bog water table are strongly affected by N emissions from oil sand operations at this time. Although N concentrations in porewater at the top of the water table were correlated with NH_4_^+^-N and NO_3_^−^-N concentrations in deposition, and porewater NH_4_^+^-N and DON concentrations decreased exponentially with distance from the oil sands industrial center (with low *R*^2^ values), the latter pattern was driven by high porewater concentrations at one site, JPH4. We did not observe an increased in bog porewater NH_4_^+^-N, NO_3_^−^-N, or DON concentrations over time, which would have been expected given the substantial increase in oil sands N emissions from 2009 to 2019.

In one of the 4 years where we examined porewater chemistry to a depth of 1 m (2011), we found high concentrations of NH_4_^+^-N, NO_3_^−^-N, and to a lesser extent DON that are atypical of Alberta bogs. Higher than usual N mineralization rates in peat in a warmer and drier growing season preceded by a drier than normal winter could have contributed to these anomalously high porewater N concentrations. It is also possible that the dry conditions led to a hydraulic reversal such that the bog porewaters became affected by groundwater more so than is typical of Alberta bogs. We cannot offer a definitive mechanistic biogeochemical explanation for the high porewater N concentrations across all bog sites in this one growing season.

Evidence that porewater SO_4_^2−^-S concentrations at the top of the bog water table may be affected by S emissions from oil sands operations was mixed. Bog porewater SO_4_^2−^-S concentrations generally decreased with distance from the oil sands industrial center (albeit largely driven by high concentrations at Mildred, which may be related to saline groundwater interactions with porewater), and were positively correlated with SO_4_^2−^-S concentrations in growing season rainfall and SO_4_^2−^-S deposition. However, given the considerable decrease in S emissions from 2009 to 2019, we would have expected a decrease in porewater SO_4_^2−^-S concentrations over time, which we did not observe. Current SO_4_^2−^-S deposition rates are at least adequate to meet the S demands for growing *Sphagnum*. Increasing S deposition may lead to downward movement of SO_4_^2−^-S into the peat, with potential consequences for CO_2_ and CH_4_ emission from bogs.

## Supplementary information

Below is the link to the electronic supplementary material.

Supplementary file1 (PDF 1275 KB)

## Data Availability

Data are available through the Environmental Data Initiative (https://doi.org/10.6073/pasta/7fb396ede46b115aae96b3d7cd1cfd20).
