# Supplementary material for: Is bog water chemistry affected by increasing N and S deposition from oil sands development in Northern Alberta, Canada?
Source: Environ Monit Assess. 2021 Nov 3;193(12):766. doi: 10.1007/s10661-021-09555-4 (PMC8566411; doi:10.1007/s10661-021-09555-4)
Supplement: Supplementary file 1 — Supplementary file1 (PDF 1275 KB) [file 10661_2021_9555_MOESM1_ESM.pdf]

Supplementary Information

Authors: R. Kelman Wieder, Melanie A. Vile, Kimberli D. Scott, James C. Quinn, Cara M. Albright, Kelly J. McMillen, Caitlyn Herron, and Hope Fillingim

Title: Is bog water chemistry affected by increasing N and S deposition from oil sands development in northern Alberta, Canada?

Journal: *Environmental Monitoring and Assessment*

Table S1. Nitrogen oxides (as NO_2) emissions (metric tonnes yr^{-1}) from oil sands facilities

Table S2. Sulfur dioxide (SO_2) emissions (metric tonnes yr^{-1}) from oil sands facilities

Table S3. Mean daily water year temperature (MAT, °C) and total precipitation (TAP, mm yr^{-1}) for each site and each water year

Table S4. *p* values from repeated measures ANOVA for water chemical parameters, 2009-2012

Table S5. Site by year interaction for porewater NH_4^+-N concentrations (μg N L^{-1}), 2009-2012; means ± standard errors.

Table S6. Site by depth interaction for porewater NH_4^+-N concentrations (μg N L^{-1}), 2009-2012

Table S7. Site by year interaction for porewater NO_3^--N concentrations (μg N L^{-1}), 2009-2012

Table S8. Year by depth interaction for porewater NO_3^--N concentrations (μg N L^{-1}), 2009-2012

Table S9. Site by year interaction for porewater DON concentrations (μg N L^{-1}), 2009-2012

Table S10. Site by depth interaction for porewater DON concentrations (μg N L^{-1}), 2009-2012

Table S11. Year by depth interaction for porewater DON concentrations (μg N L^{-1}), 2009-2012

Table S12. Site by year by depth interaction for porewater DON/TDN concentrations (%), 2009-2012

Table S13. Site by year by depth interaction for porewater DOC concentrations (mg C L^{-1}), 2009-2012

Table S14. Site by year by depth interaction for porewater SO_4^{2-}-S concentrations (μg S L^{-1}), 2009-2012

Fig. S1. Wind roses generated for the 12-month periods 1 May through 30 April

Fig. S2. Interpolated porewater reduced conductivity at 5 peatland sites as a function of time and depth

---

Reference for Tables S1 and S1: CEMA. (2012). Development of a modeling emissions inventory database for the implementation of emissions management framework. Retrieved from Cumulative Environmental Management Association: http://library.cemaonline.ca/ckan/dataset/2011-0038

Table S1. Nitrogen oxides (as NO_2) emissions (metric tonnes yr^{-1}) from oil sands facilities. NPRI emissions are those reported to the Environment Canada National Pollutant Release Inventory (https://www.canada.ca/en/services/environment/pollution-waste-management/national-pollutant-release-inventory.html). As the NPRI does not capture emissions from mine fleets, these were calculated from an NO_2 emission factor based on annual crude bitumen production (0.7804 kg NO_2/m^3 of bitumen production; CEMA 2012). Crude bitumen production data were obtained from Alberta Mineable Oil Sands Plant Statistics, Monthly Supplement, December of each year (https://www.aer.ca/providing-information/data-and-reports/statistical-reports/st39). Data from facilities whose emissions never exceeded 100 tonnes/yr have not been included.

	Syncrude Mildred Lake (2274)		Suncor Base Plant (2230)		CNRL Horizon (23275)		Syncrude Aurora (6572)		Nexen Long Lake (22378)	Shell Muskeg River Jackpine Mines (6647)		Imperial Oil Kearl Sands Mine (27506)		Suncor MacKay River (17630)	Suncor Firebag (19181)	
Year	NPRI	Fleet	NPRI	Fleet	NPRI	Fleet	NPRI	Fleet	NPRI	NPRI	Fleet	NPRI	Fleet	NPRI	NPRI	Total
1998	12936	11425	3957	5119												33437
1999	13860	12351	4205	5913												36329
2000	12131	10408	4733	6469												33741
2001	11229	9596	4712	6940				1688								34164
2002	11963	8893	8830	12217			288	3618								45521
2003	11488	7870	9011	12259			310	4004		345	27			20		45025
2004	11410	6613	11104	12283			455	6858		518	12341			58	131	61317
2005	11603	5670	10901	8864			489	6696		732	8907			43	185	53601
2006	14617	5722	9990	13761			631	8985		469	13797			36	397	67775
2007	15058	7313	10520	12088			671	10001		652	12131			43	545	68351
2008	12732	7451	9842	11259			687	8579	1094	641	11322			63	553	63535
2009	13932	6544	11693	13110	1760	2800	575	9160	1653	649	13233			123	664	75321
2010	14074	6950	11359	12047	1923	4950	548	9419	2776	744	12115			68	963	77388
2011	15701	6745	11765	13032	1115	2178	643	9602	2179	979	13089			57	1856	78297
2012	13578	6887	10490	12088	1606	4671	557	8904	1793	1043	12214			126	1902	75302
2013	14120	6692	7920	12215	1532	5305	608	8707	3389	1247	12269	259	1131	54	2448	77287
2014	14453	5928	8501	12425	1383	5878	660	8789	3149	1279	12470	319	3605	45	2611	80836
2015	14894	8177	9341	13928	1668	6592	630	8810	2409	1087	13928	595	7595	48	2563	91684
2016	13885	6286	7797	10812	2085	6644	713	9005	1676	1191	10812	617	8416	59	2450	81793
2017	14014	5975	8980	13800	2587	8910	692	8386	1959	1270	13800	657	8883	60	2469	91810
2018	13988	5358	8862	11714	2734	11949	590	8907	1827	1228	11773	638	10083	59	2891	92011
2019	13318	6535	13080	13122	3027	8983	589	10102	1671	1014	13247	713	9992	125	2944	97873

Table S2. Sulfur dioxide (SO_2) emissions (metric tonnes yr^{-1}) from oil sands facilities. NPRI emissions are those reported to the Environment Canada National Pollutant Release Inventory (https://www.canada.ca/en/services/environment/pollution-waste-management/national-pollutant-release-inventory.html). As the NPRI does not capture emissions from mine fleets, these were calculated from an SO_2 emission factor based on annual crude bitumen production (0.0150 kg SO_2/m^3 of bitumen production; CEMA 2012). Crude bitumen production data were obtained from Alberta Mineable Oil Sands Plant Statistics, Monthly Supplement, December of each year (https://www.aer.ca/providing-information/data-and-reports/statistical-reports/st39). Data from facilities whose emissions never exceeded 100 tonnes/yr have not been included.

	Syncrude Mildred Lake (2274)		Suncor Base Plant (2230)		CNRL Horizon (23275)		Nexen Long Lake (22378)	Shell Muskeg River Jackpine Mines (6647)	Imperial Oil Kearl Sands Mine (27506)	Suncor MacKay River (17630)	Suncor Firebag (19181)	Syncrude Aurora (6572)	
Year	NPRI	Fleet	NPRI	Fleet	NPRI	Fleet	NPRI	Fleet	Fleet	NPRI	NPRI	NPRI	Total
1998	88403	219	9868	98									98588
1999	94713	237	10487	113									105550
2000	82897	200	11802	124									95023
2001	76734	184	11750	133								32	88834
2002	81572	171	22020	234								64	104061
2003	78240	151	18663	235				75		24		71	97459
2004	78322	127	25100	236				127		47	238	123	104319
2005	83988	109	25779	170				146		68	881	119	111260
2006	80863	110	24118	264				126		82	1256	160	107040
2007	83973	140	26645	232				131		73	1075	179	112448
2008	70538	143	28997	216				117		82	840	151	101085
2009	81471	126	19027	251	10572	54	7151	121		112	1002	165	120052
2010	72769	133	22210	231	2453	95	3222	115		48	1140	170	102587
2011	64727	129	20258	250	1988	42	1743	182		106	369	172	89966
2012	72971	132	18539	232	2423	90	3076	196		179	348	160	98346
2013	63132	128	14104	234	4073	102	2878	206	22	152	347	155	85512
2014	25561	114	16683	238	2995	113	2329	212	69	111	412	156	48923
2015	29046	157	12642	267	3054	126	1710	207	146		211	157	47370
2016	22555	121	12955	207	3409	127	215	223	161		273	159	40022
2017	28731	115	12695	265	2640	171	220	238	170		148	148	45132
2018	31581	103	13632	225	2696	229	270	256	193	174	241	160	49310
2019	27673	125	13402	252	2834	172	242	251	192	183	346	183	45412

Table S3. Mean daily water year temperature (MAT, ^oC) and total precipitation (TAP, mm yr^{-1}) for each site and each water year (November-October). There were significant differences between sites for both MAT and TAP ($p < 0.0001$; ANOVA with site as the main effect and water year as a blocked effect; *a posteriori* means comparisons using Tukey's Honestly Significant Difference test. Means with the same superscript letter do not differ significantly (Tukey's Honestly Significant difference test).

			Site						
Parameter	Water Year	Water year average	Mildred	JPH4	Kearl	McKay	McMurray	Horse Creek	Anzac
Daily water year temperature (^oC)	1989-1990	-0.2	-0.3	-0.8	-0.9	-0.5	0.3	0.8	0.3
	1990-1991	-0.1	-0.2	-0.7	-0.7	-0.5	0.4	0.5	0.3
	1991-1992	0.7	0.6	0.2	0.1	0.3	1.3	1.4	1.1
	1992-1993	0.7	0.6	0.4	0.4	0.4	1.1	0.9	0.9
	1994-1994	0.9	1.2	0.8	0.6	0.7	1.2	1.2	1.0
	1994-1995	-1.1	1.1	0.8	0.6	0.7	1.1	0.9	0.8
	1995-1996	0.0	-1.0	-1.1	-1.3	-1.3	-0.9	-1.2	-1.2
	1996-1997	3.4	0.2	-0.1	-0.3	-0.2	0.3	0.0	0.0
	1997-1998	1.8	3.7	3.4	3.2	3.3	3.6	3.5	3.4
	1998-1999	1.5	2.2	1.8	1.6	1.6	2.1	1.7	1.7
	1999-2000	1.7	1.8	1.4	1.1	1.2	1.8	1.6	1.5
	2000-2001	-0.3	2.0	1.5	1.3	1.4	2.0	1.8	1.7
	2001-2002	1.2	0.1	-0.4	-0.7	-0.6	0.0	-0.1	-0.2
	2002-2003	0.1	1.5	1.1	0.8	0.9	1.5	1.4	1.2
	2003-2004	1.1	0.5	0.0	-0.2	-0.1	0.3	0.2	0.1
	2004-2005	3.5	1.6	1.0	0.7	0.9	1.2	1.4	1.1
	2005-2006	0.9	4.0	3.6	3.4	3.5	3.5	3.4	3.4
	2006-2007	0.6	1.3	0.8	0.6	0.8	0.8	1.0	0.8
	2007-2008	0.0	1.1	0.7	0.4	0.5	0.4	0.6	0.4
	2008-2009	2.5	0.4	0.0	-0.2	-0.2	0.0	0.1	-0.1
	2009-2010	1.1	3.0	2.7	2.5	2.5	2.5	2.3	2.4
	2010-2011	2.8	1.7	1.3	0.9	1.1	0.9	0.9	0.8
	2011-2012	0.9	3.3	2.9	2.5	2.7	2.6	2.7	2.5
	2012-2013	-0.4	1.4	1.0	0.6	0.8	0.9	0.7	0.7
	2013-2014	1.6	0.3	-0.2	-0.6	-0.3	-0.5	-0.7	-0.7
	2014-2015	3.1	2.3	1.8	1.4	1.7	1.5	1.4	1.3
	2015-2016	2.5	3.6	3.1	2.8	3.0	3.2	2.8	3.0
	2016-2017	0.2	3.1	2.6	2.4	2.4	2.5	2.0	2.2
	2017-2018	0.4	0.9	0.3	0.1	0.1	0.2	-0.2	-0.1
	2018-2019	1.2	1.2	0.5	0.3	0.2	0.6	-0.2	0.3
	30-year mean	1.1	1.4^{a}	1.0^{cd}	0.8^{e}	0.9^{de}	1.2^{b}	1.1^{bc}	1.0^{b-d}

Total precipitation (mm)	1989-1990	438	470	426	429	421	454	443	422
	1990-1991	529	509	479	477	476	589	589	586
	1991-1992	449	451	437	432	437	429	451	504
	1992-1993	438	397	405	435	407	398	507	514
	1994-1994	411	377	397	445	418	340	419	461
	1994-1995	521	497	507	518	519	478	547	581
	1995-1996	600	567	567	562	570	607	669	656
	1996-1997	514	493	512	538	532	479	509	534
	1997-1998	282	209	242	279	286	237	386	333
	1998-1999	370	292	323	354	358	351	432	484
	1999-2000	461	385	424	493	478	440	494	509
	2000-2001	446	370	389	402	405	417	569	571
	2001-2002	458	408	420	440	434	438	485	581
	2002-2003	507	445	461	478	450	511	544	661
	2003-2004	392	362	372	392	389	307	460	458
	2004-2005	542	508	510	518	526	468	613	652
	2005-2006	432	411	412	411	433	374	467	518
	2006-2007	406	354	387	435	431	289	464	485
	2007-2008	464	401	444	500	456	406	526	517
	2008-2009	380	274	302	351	317	416	479	520
	2009-2010	408	401	399	392	410	360	422	477
	2010-2011	352	241	262	300	295	432	423	509
	2011-2012	570	471	512	617	495	541	619	732
	2012-2013	513	456	476	535	487	410	623	606
	2013-2014	452	378	399	437	420	431	526	573
	2014-2015	332	305	318	357	322	301	376	346
	2015-2016	515	442	471	532	476	532	548	601
	2016-2017	331	309	293	298	305	281	420	408
	2017-2018	426	408	420	420	426	389	470	451
	2018-2019	420	373	404	413	402	414	490	444
	30-year mean	445	399 [c]	412 [bc]	440 [b]	426 [bc]	417 [bc]	500 [a]	523 [a]

Table S4. *p* values from repeated measures ANOVA for water chemical parameters, 2009-2012.

	Parameter					
Effect	NH_4^+-N (μg N L^{-1})	NO_3^--N (μg N L^{-1})	DON (mg N L^{-1})	DON/TDN	DOC (mg C L^{-1})	SO_4^2 -S (μg S L^{-1})
Site	<0.0001	0.0012	<0.0001	<0.0001	<0.0001	<0.0001
Year	<0.0001	<0.0001	<0.0001	<0.0001	<0.0001	0.0011
Depth	<0.0001	0.0130	<0.0001	<0.0001	<0.0001	<0.0001
Site x Year	0.0575	<0.0001	<0.0001	<0.0001	<0.0001	0.0008
Site x Depth	<0.0001	0.1511	<0.0001	<0.0001	<0.0001	<0.0001
Year x Depth	0.2785	0.0119	<0.0001	0.0001	<0.0001	0.0004
Site x Year x Depth	0.9929	0.2559	0.1546	0.0023	0.0066	0.0074

Table S5. Site by year interaction for porewater NH_4^+-N concentrations (μg N L^{-1}), 2009-2012; means ± standard errors.

	Site				
Year	Mildred	JPH4	McKay	McMurray	Anzac
2009	225 ± 77	-	771 ± 89	256 ± 16	622 ± 42
2010	90 ± 5	1727 ± 191	475 ± 62	292 ± 17	695 ± 36
2011	1041 ± 87	3753 ± 168	1731 ± 85	1264 ± 21	1709 ± 49
2012	237 ± 64	1718 ± 155	687 ± 95	177 ± 11	605 ± 42

Table S6. Site by depth interaction for porewater NH_4^+-N concentrations (μg N L^{-1}), 2009-2012; means ± standard errors.

	Site				
Depth	Mildred	JPH4	McKay	McMurray	Anzac
0-10	-	-	140 ± 50	-	100 ± 26
10-20	132 ± 22	470 ± 312	357 ± 85	160 ± 29	206 ± 53
20-30	298 ± 83	194 ± 57	493 ± 71	295 ± 43	338 ± 55
30-40	361 ± 75	1270 ± 208	649 ± 78	405 ± 55	442 ± 55
40-50	485 ± 114	2504 ± 227	703 ± 80	493 ± 58	575 ± 57
50-60	264 ± 106	2606 ± 280	955 ± 107	449 ± 55	723 ± 60
60-70	236 ± 82	3173 ± 302	1047 ± 124	580 ± 62	1026 ± 66
70-80	355 ± 95	3190 ± 262	1128 ± 142	602 ± 63	1291 ± 63
80-90	445 ± 163	3608 ± 273	1269 ± 166	594 ± 56	1513 ± 62
90-100	483 ± 190	3872 ± 252	1518 ± 189	706 ± 58	1715 ± 61

Table S7. Site by year interaction for porewater NO_3^--N concentrations (μg N L^{-1}), 2009-2012; means ± standard errors.

	Site				
Year	Mildred	JPH4	McKay	McMurray	Anzac
2009	19 ± 3	-	23 ± 5	58 ± 30	153 ± 2
2010	5 ± 1	12 ± 2	9 ± 6	12 ± 1	55 ± 1
2011	192 ± 20	411 ± 49	218 ± 10	133 ± 6	250 ± 13
2012	47 ± 10	86 ± 12	26 ± 2	37 ± 6	16 ± 3

Table S8. Year by depth interaction for porewater NO_3^--N concentrations (μg N L^{-1}), 2009-2012; means ± standard errors.

	Year			
Depth	2009	2010	2011	2012
0-10	30	0 ± 0	-	10
10-20	34 ± 8	49 ± 41	95 ± 21	21 ± 5
20-30	47 ± 28	5 ± 2	203 ± 24	31 ± 7
30-40	15 ± 2	8 ± 1	211 ± 15	37 ± 6
40-50	13 ± 2	6 ± 1	218 ± 15	43 ± 9
50-60	20 ± 6	5 ± 1	227 ± 16	37 ± 10
60-70	12 ± 2	8 ± 1	226 ± 20	40 ± 9
70-80	19 ± 6	8 ± 2	216 ± 19	29 ± 6
80-90	21 ± 4	7 ± 1	237 ± 23	25 ± 4
90-100	107 ± 73	7 ± 1	260 ± 34	44 ± 9

Table S9. Site by year interaction for porewater DON concentrations (μg N L^{-1}), 2009-2012; means ± standard errors.

	Site				
Year	Mildred	JPH4	McKay	McMurray	Anzac
2009	1942 ± 137	-	2564 ± 133	3348 ± 153	2329 ± 108
2010	1011 ± 34	4585 ± 411	2082 ± 61	2427 ± 74	1856 ± 56
2011	1178 ± 56	6492 ± 346	2757 ± 72	3044 ± 67	2412 ± 58
2012	1129 ± 115	3517 ± 253	1826 ± 71	1898 ± 61	1308 ± 53

Table S10. Site by depth interaction for porewater DON concentrations (μg N L^{-1}), 2009-2012; means ± standard errors.

	Site				
Depth	Mildred	JPH4	McKay	McMurray	Anzac
0-10	-	-	1170 ± 40	-	1357 ± 405
10-20	1246 ± 219	2746 ± 749	1483 ± 116	1257 ± 142	864 ± 74
20-30	1405 ± 143	2086 ± 207	1621 ± 78	1482 ± 57	976 ± 50
30-40	1347 ± 98	3479 ± 310	1885 ± 71	1599 ± 61	1166 ± 52
40-50	1412 ± 146	4225 ± 467	2036 ± 80	1926 ± 65	1363 ± 54
50-60	1220 ± 105	5551 ± 656	2232 ± 109	2462 ± 90	1739 ± 68
60-70	1162 ± 95	5332 ± 592	2574 ± 116	3043 ± 104	2250 ± 75
70-80	1027 ± 118	6895 ± 972	2619 ± 144	3319 ± 123	2719 ± 93
80-90	1271 ± 181	6344 ± 515	2734 ± 147	3589 ± 131	2678 ± 96
90-100	1202 ± 220	6381 ± 469	2876 ± 147	3977 ± 155	2779 ± 103

Table S11. Year by depth interaction for porewater DON concentrations (μg N L^{-1}), 2009-2012; means ± standard errors. Note: only one sample from the 0-10 cm depth in 2009.

	Year			
Depth	2009	2010	2011	2012
0-10	208	1217 ± 52	-	680
10-20	1329 ± 161	1338 ± 253	1301 ± 147	1159 ± 162
20-30	1341 ± 76	1287 ± 68	1720 ± 76	1228 ± 99
30-40	1585 ± 87	1441 ± 81	2186 ± 141	1455 ± 93
40-50	1981 ± 120	1656 ± 93	2446 ± 163	1532 ± 110
50-60	2496 ± 140	2052 ± 112	3012 ± 227	1950 ± 247
60-70	2941 ± 167	2415 ± 121	3141 ± 177	2088 ± 126
70-80	3510 ± 205	2690 ± 200	3160 ± 164	2130 ± 143
80-90	3705 ± 201	2776 ± 148	3533 ± 204	2214 ± 140
90-100	4197 ± 281	2979 ± 110	3907 ± 202	2551 ± 154

Table S12. Site by year by depth interaction for porewater DON/TDN concentrations (%), 2009-2012; means ± standard errors. Values without standard errors have a sample size of 1.

		Site				
Depth	Year	Mildred	JPH4	McKay	McMurray	Anzac
0-10	2009	-	-	-	-	94.5
	2010	-	-	89.5 ± 3.1	-	89.7
	2011	-	-	-	-	-
	2012	-	-	-	-	90.7
10-20	2009	87.8 ± 2.8	-	90.5 ± 2.9	80.1 ± 6.2	92.7 ± 1.7
	2010	89.3 ± 1.2	98.1	76.9 ± 12.1	84.3 ± 3.2	80.4 ± 3.8
	2011	85.2 ± 1.1	77.8	73.5 ± 5.1	-	54.2 ± 6.3
	2012	97.8	93.2 ± 1.6	95.3 ± 2.4	90.6 ± 2.9	83.4 ± 5.0
20-30	2009	91.9 ± 2.1	-	84.5 ± 3.5	88.9 ± 1.7	92.9 ± 1.6
	2010	89.2 ± 1.4	87.5 ± 5.4	88.8 ± 1.6	86.0 ± 2.0	86.2 ± 1.8
	2011	61.6 ± 8.3	-	63.0 ± 2.4	64.3 ± 1.6	53.7 ± 2.9
	2012	90.2 ± 5.4	93.0 ± 1.2	89.7 ± 1.2	91.3 ± 1.3	83.3 ± 1.9
30-40	2009	91.0 ± 1.6	-	83.4 ± 4.2	91.6 ± 1.1	89.9 ± 1.7
	2010	88.5 ± 1.3	78.9 ± 4.8	88.3 ± 2.0	87.5 ± 2.9	83.7 ± 1.6
	2011	52.8 ± 4.1	63.0 ± 1.9	62.2 ± 2.6	62.1 ± 0.8	53.7 ± 1.5
	2012	87.9 ± 5.8	79.5 ± 2.1	86.3 ± 2.4	91.4 ± 1.1	81.3 ± 2.1
40-50	2009	94.2 ± 1.2	-	82.4 ± 5.5	91.6 ± 1.1	83.6 ± 1.6
	2010	92.1 ± 1.2	65.5 ± 2.3	87.6 ± 2.2	88.9 ± 1.8	79.4 ± 1.8
	2011	56.6 ± 5.3	58.5 ± 2.0	62.5 ± 2.3	63.1 ± 0.8	53.0 ± 1.2
	2012	83.3 ± 6.1	58.1 ± 5.6	85.8 ± 3.2	89.1 ± 2.0	77.2 ± 2.1
50-60	2009	95.0 ± 1.1	-	78.8 ± 5.9	92.6 ± 0.8	83.5 ± 1.7
	2010	92.1 ± 1.0	70.1 ± 7.0	87.8 ± 2.2	89.4 ± 1.5	78.5 ± 1.9
	2011	61.0 ± 10.4	60.5 ± 2.2	60.0 ± 2.4	66.6 ± 0.8	53.4 ± 1.1
	2012	92.3 ± 3.1	66.8 ± 3.7	82.2 ± 3.6	89.8 ± 0.9	73.9 ± 2.2
60-70	2009	94.0 ± 1.3	-	80.3 ± 6.1	93.6 ± 1.1	77.7 ± 1.3
	2010	91.8 ± 1.9	51.6 ± 20.0	87.1 ± 3.2	89.8 ± 1.4	76.0 ± 1.4
	2011	58.1 ± 7.6	58.5 ± 2.0	63.4 ± 2.6	68.6 ± 0.8	54.2 ± 2.1
	2012	94.3 ± 1.5	63.0 ± 1.2	81.6 ± 4.3	88.8 ± 1.3	68.3 ± 1.3
70-80	2009	83.0 ± 4.0	-	86.5 ± 4.1	93.4 ± 0.9	77.3 ± 1.2
	2010	91.2 ± 2.3	66.7 ± 7.9	87.7 ± 3.2	90.1 ± 1.1	71.9 ± 1.3
	2011	52.2 ± 8.8	66.7 ± 2.6	62.6 ± 2.4	69.8 ± 0.8	54.9 ± 0.7
	2012	83.8 ± 8.2	58.4 ± 0.7	79.3 ± 4.8	90.6 ± 1.1	66.4 ± 1.1
80-90	2009	95.5 ± 1.0	-	85.1 ± 4.6	92.8 ± 0.9	72.0 ± 1.4
	2010	93.8 ± 1.5	74.2 ± 2.7	87.5 ± 3.6	89.8 ± 1.1	67.4 ± 1.3
	2011	46.6 ± 10.4	58.9 ± 1.6	61.0 ± 2.6	73.0 ± 0.7	52.0 ± 1.2
	2012	59.5 ± 7.1	56.1 ± 4.1	81.0 ± 4.6	89.8 ± 1.6	62.6 ± 0.5
90-100	2009	-	-	82.1 ± 4.5	89.2 ± 3.8	69.6 ± 1.8
	2010	97.5 ± 0.5	60.3 ± 6.5	85.6 ± 4.2	87.8 ± 1.4	64.3 ± 1.2
	2011	48.9	60.7 ± 3.0	57.1 ± 1.9	73.4 ± 0.5	51.7 ± 0.7
	2012	46.5 ± 5.5	58.1 ± 1.8	78.0 ± 5.0	88.4± 1.3	58.7 ± 0.9

Table S13. Site by year by depth interaction for porewater DOC concentrations (mg C L^{-1}), 2009-2012; means ± standard errors. Values without standard errors have a sample size of 1.

		Site				
Depth	Year	Mildred	JPH4	McKay	McMurray	Anzac
0-10	2009	-	-	-	-	44.9
	2010	-	-	41.5 ± 12.4	-	50.2
	2011	-	-	-	-	-
	2012	-	-	-	-	46.6
10-20	2009	63.5 ± 8.7	-	64.7 ± 5.6	79.2 ± 10.0	45.7 ± 2.4
	2010	44.3 ± 2.7	86.1	69.5 ± 5.0	74.2 ± 7.2	43.9 ± 4.8
	2011	29.0 ± 7.3	57.3	63.4 ± 2.5	-	33.8 ± 5.9
	2012	72.6	105.8 ± 16.7	66.4 ± 3.6	76.2 ± 10.1	40.0 ± 3.6
20-30	2009	52.3 ± 3.3	-	74.1 ± 5.6	86.3 ± 2.8	46.9 ± 1.8
	2010	29.9 ± 5.7	84.9 ± 5.7	65.4 ± 3.6	77.6 ± 3.2	46.0 ± 3.2
	2011	40.3 ± 0.6	-	66.1 ± 2.1	84.2 ± 1.6	43.8 ± 1.9
	2012	56.4 ± 5.4	96.0 ± 7.5	59.1 ± 5.5	87.0 ± 4.4	44.5 ± 2.7
30-40	2009	47.5 ± 4.4	-	80.7 ± 4.3	89.0 ± 2.3	50.7 ± 2.1
	2010	28.8 ± 2.4	97.5 ± 4.2	76.2 ± 2.6	79.5 ± 4.0	46.4 ± 2.5
	2011	30.8 ± 2.3	105.2 ± 8.5	67.0 ± 3.1	85.3 ± 2.5	46.5 ± 1.4
	2012	49.4 ± 4.1	97.1 ± 9.8	73.3 ± 3.0	89.8 ± 3.9	47.3 ± 1.7
40-50	2009	53.9 ± 5.4	-	86.0 ± 3.7	106.4 ± 3.6	52.9 ± 2.6
	2010	30.4 ± 3.5	117.4 ± 4.3	74.9 ± 3.2	89.2 ± 3.6	52.5 ± 2.0
	2011	26.7 ± 2.4	128.5 ± 12.8	75.0 ± 1.7	91.5 ± 4.2	49.7 ± 1.0
	2012	37.0 ± 4.5	106.7 ± 10.1	75.4 ± 2.3	102.1 ± 4.4	51.2 ± 1.0
50-60	2009	46.0 ± 3.3	-	82.7 ± 3.9	154.3 ± 12.3	59.7 ± 2.0
	2010	29.5 ± 8.5	112.4 ± 4.0	74.0 ± 3.0	117.0 ± 3.8	54.4 ± 2.5
	2011	32.8 ± 1.7	118.6 ± 10.3	75.7 ± 2.7	110.3 ± 3.9	52.3 ± 1.0
	2012	40.2 ± 3.2	103.6 ± 13.2	78.5 ± 2.7	117.8 ± 4.5	48.9 ± 2.6
60-70	2009	38.1 ± 3.7	-	94.5 ± 4.1	182.7 ± 9.9	64.8 ± 1.7
	2010	21.0 ± 6.0	130.0 ± 8.3	83.5 ± 2.9	144.8 ± 6.4	61.6 ± 2.1
	2011	27.4 ± 2.9	110.1 ± 3.4	80.9 ± 1.7	135.1 ± 4.9	58.7 ± 0.8
	2012	51.2 ± 3.3	90.7 ± 7.6	76.3 ± 3.0	140.2 ± 6.1	58.0 ± 1.4
70-80	2009	24.9 ± 2.0	-	90.8 ± 4.0	187.7 ± 11.1	68.2 ± 2.2
	2010	19.3 ± 5.8	121.3 ± 13.5	77.3 ± 3.1	162.9 ± 7.1	63.3 ± 2.8
	2011	24.3 ± 1.3	118.7 ± 7.7	80.9 ± 1.7	151.2 ± 5.9	62.0 ± 0.9
	2012	32.0 ± 3.6	90.2 ± 5.1	73.1 ± 4.0	146.8 ± 7.3	60.2 ± 1.5
80-90	2009	27.3 ± 1.2	-	98.7 ± 4.3	228.5 ± 12.1	70.6 ± 2.5
	2010	12.4 ± 4.7	116.3 ± 6.1	82.1 ± 2.8	189.3 ± 7.3	61.4 ± 2.9
	2011	16.7 ± 1.5	119.2 ± 4.7	79.5 ± 1.8	180.6 ± 6.4	62.0 ± 1.4
	2012	25.7 ± 2.2	85.6 ± 3.9	73.2 ± 4.7	169.3 ± 8.6	61.2 ± 2.9
90-100	2009	-	-	95.2 ± 4.8	258.1 ± 13.1	70.8 ± 2.9
	2010	13.3 ± 0.5	114.1 ± 1.1	82.9 ± 2.2	203.9 ± 5.2	64.6 ± 2.7
	2011	16.9	117.2 ± 8.6	82.5 ± 1.5	192.8 ± 3.9	63.1 ± 1.7
	2012	15.5 ± 1.1	91.9 ± 3.4	76.6 ± 4.3	191.2 ± 6.2	62.9 ± 2.9

Table S14. Site by year by depth interaction for porewater SO_4^{2-}-S concentrations (µg S L^{-1}), 2009-2012; means ± standard errors. Values without standard errors have a sample size of 1.

		Site				
Depth	Year	Mildred	JPH4	McKay	McMurray	Anzac
0-10	2009	-	-	-	-	510
	2010	-	-	435 ± 215	-	140
	2011	-	-	-	-	-
	2012	-	-	-	-	230
10-20	2009	4645 ± 665	-	625 ± 118	105 ± 43	177 ± 59
	2010	8877 ± 2345	1200	688 ± 173	97 ± 74	141 ± 20
	2011	3175 ± 2045	1330	478 ± 120	-	95 ± 34
	2012	12040	1037 ± 262	285 ± 105	144 ± 43	80 ± 15
20-30	2009	13562 ± 7440	-	247 ± 54	70 ± 13	85 ± 22
	2010	9708 ± 1545	1164 ± 208	309 ± 118	58 ± 19	58 ± 7
	2011	6422 ± 934	-	197 ± 71	38 ± 14	16 ± 3
	2012	11704 ± 2951	692 ± 239	125 ± 40	241 ± 134	28 ± 12
30-40	2009	7017 ± 2339	-	504 ± 197	64 ± 13	69 ± 15
	2010	7982 ± 1251	633 ± 227	340 ± 155	35 ± 5	42 ± 5
	2011	6394 ± 740	435 ± 136	534 ± 238	26 ± 5	10 ± 1
	2012	11659 ± 2102	1204 ± 369	181 ± 88	79 ± 27	29 ± 10
40-50	2009	2243 ± 736	-	152 ± 30	62 ± 13	63 ± 10
	2010	6636 ± 682	193 ± 119	230 ± 82	29 ± 3	32 ± 5
	2011	5236 ± 464	384 ± 201	87 ± 39	13 ± 1	7 ± 1
	2012	8345 ± 1427	1044 ± 253	152 ± 58	48 ± 21	19 ± 8
50-60	2009	14068 ± 9918	-	411 ± 154	57 ± 11	61 ± 11
	2010	10802 ± 1415	327 ± 155	309 ± 91	23 ± 2	33 ± 6
	2011	5540 ± 751	467 ± 118	412 ± 157	12 ± 1	75 ± 1
	2012	7043 ± 1355	1305 ± 483	327 ± 143	27 ± 16	25 ± 17
60-70	2009	12166 ± 8269	-	99 ± 22	69 ± 15	86 ± 30
	2010	7314 ± 497	67 ± 12	95 ± 38	25 ± 2	39 ± 6
	2011	6078 ± 486	93 ± 34	47 ± 24	12 ± 1	6 ± 1
	2012	12032 ± 1750	1120 ± 316	26 ± 7	25 ± 14	5 ± 1
70-80	2009	6006 ± 2644	-	95 ± 24	61 ± 13	71 ± 14
	2010	7043 ± 379	214 ± 89	38 ± 6	25 ± 2	38 ± 6
	2011	5180 ± 426	25 ± 5	19 ± 7	12 ± 1	8 ± 3
	2012	7926 ± 2306	707 ± 361	13 ± 3	22 ± 16	5 ± 1
80-90	2009	1360 ± 731	-	102 ± 23	71 ± 14	72 ± 14
	2010	3639 ± 881	40 ± 14	35 ± 5	29 ± 3	34 ± 6
	2011	3978 ± 611	16 ± 2	14 ± 2	12 ± 1	78 ± 2
	2012	5240 ± 1124	148 ± 70	11 ± 1	8 ± 1	5 ± 2
90-100	2009	-	-	319 ± 225	81 ± 16	70 ± 13
	2010	2270 ± 1198	80 ± 40	30 ± 5	27 ± 2	34 ± 5
	2011	2840	22 ± 12	12 ± 1	15 ± 1	6 ± 1
	2012	4330 ± 323	31 ± 9	13 ± 4	31 ± 23	5 ± 3

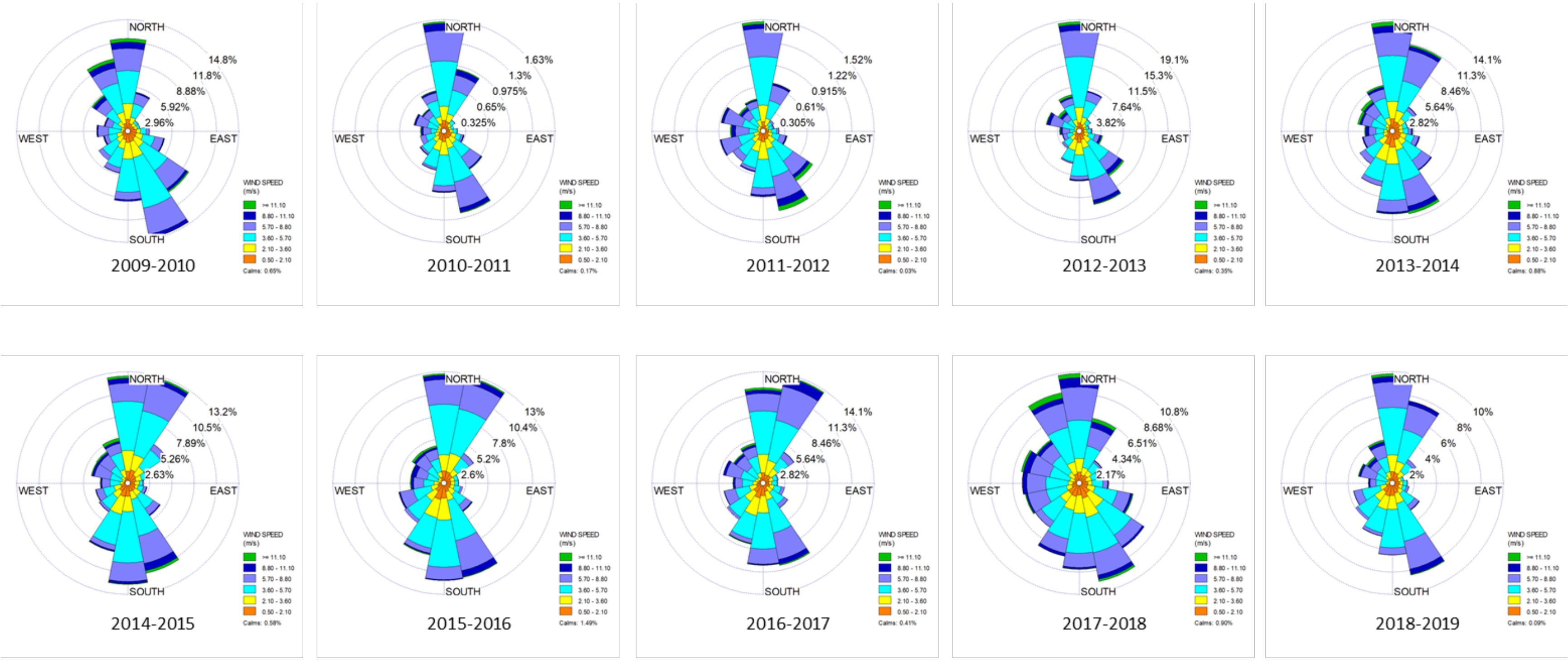

Fig. S1. Wind roses were generated for the 12-month periods 1 May through 30 April using wind speed and wind direction data from the Alberta government Mildred Lake Station (57.03ºN, 111.45ºW; https://acis.alberta.ca/weather-data-viewer.jsp) using WRPLOT View, version 8.0.2 (Lakes Environmental Software, Waterloo, Ontario, Canada).

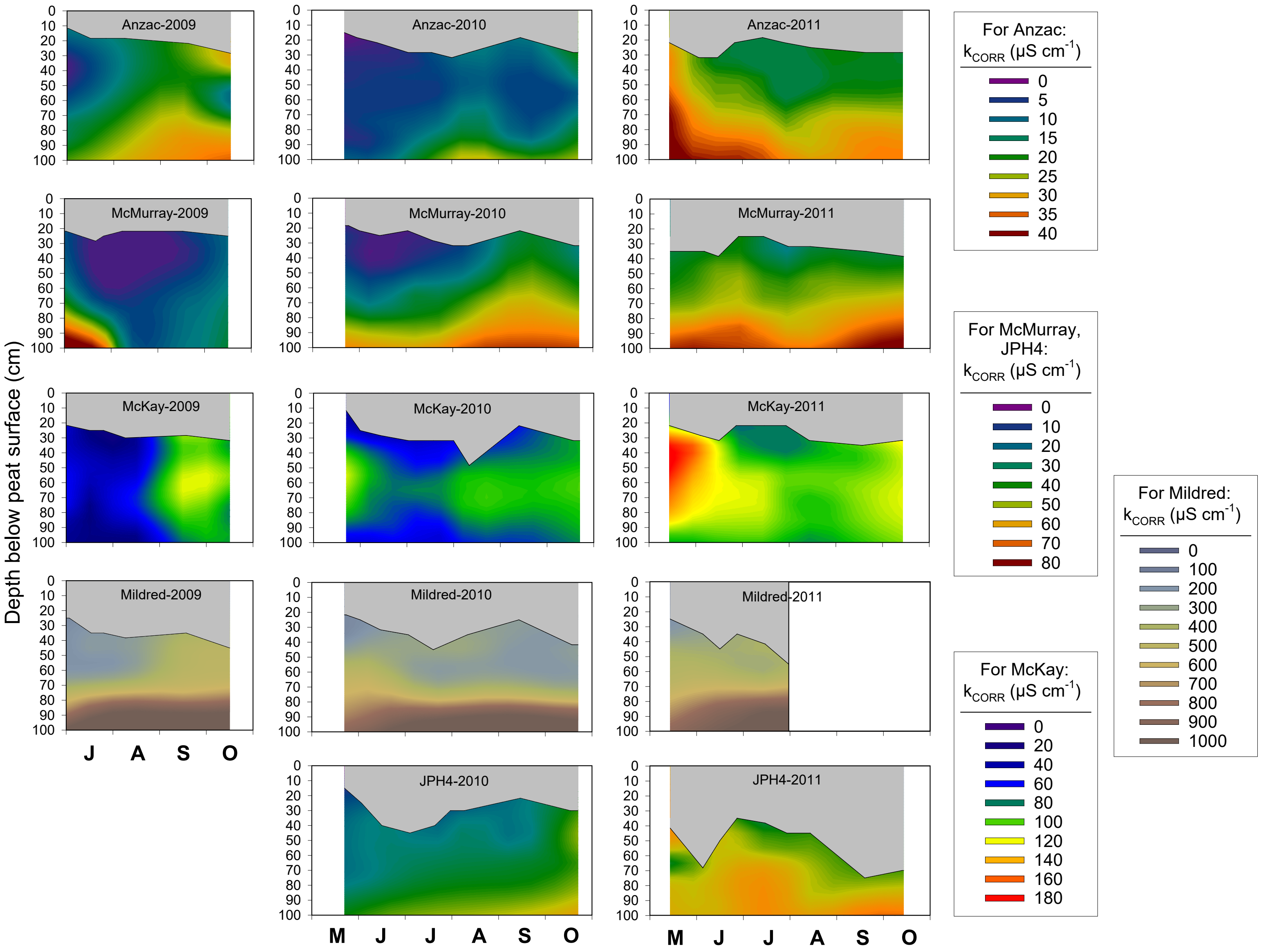

Fig. S2. Interpolated porewater reduced conductivity at 5 peatland sites as a function of time and depth. Grey areas indicate the zone of peat between the peat surface and the water table.
